# Rapid Review of COVID-19 Vaccination Access and Acceptance for Global Refugee, Asylum Seeker and Undocumented Migrant Populations

**DOI:** 10.3389/ijph.2022.1605508

**Published:** 2022-12-22

**Authors:** Ariadne A. Nichol, Zoi Parcharidi, Wael K. Al-Delaimy, Elias Kondilis

**Affiliations:** ^1^ School of Medicine, University of California, San Diego, La Jolla, CA, United States; ^2^ Center for Biomedical Ethics, School of Medicine, Stanford University, Stanford, CA, United States; ^3^ School of Medicine, Aristotle University of Thessaloniki, Thessaloniki, Greece; ^4^ Herbert Wertheim School of Public Health and Human Longevity Science, University of California, San Diego, La Jolla, CA, United States

**Keywords:** vaccine acceptance, refugee, migrant, COVID-19 vaccination, vaccination access

## Abstract

**Objectives:** Refugees, asylum seekers, and undocumented migrants globally have been disproportionally impacted by COVID-19. Vaccination has been a major tool to reduce disease impact, yet concerns exist regarding equitable allocation and uptake.

**Methods:** A rapid literature review was conducted based on PRISMA guidelines to determine COVID-19 vaccination acceptance rates and level of access for these population groups globally.

**Results:** Relatively high COVID-19 vaccine acceptance levels were commonly reported in these populations, although, trust in host governments was a frequently expressed concern, especially for undocumented migrants. Outreach efforts and access to comprehensive information from a trusted source and in appropriate language were found to be major determinants of COVID-19 vaccine acceptance. COVID-19 vaccination access and policies varied considerably across host countries despite urgings by international organizations to include migrants and refugees. While most governments endorsed inclusive policies, evidence of successful program implementation was frequently lacking, creating difficulty to better tailor and implement COVID-19 outreach programs.

**Conclusion:** This review identifies impactful improvements to be implemented to ensure equitable COVID-19 vaccinations and to reduce disease burden on refugees, asylum seekers, and undocumented migrants.

## Introduction

The COVID-19 pandemic has posed unprecedented public health challenges across the globe. According to data reported to the World Health Organization (WHO), as of 17 October 2022 there have been almost 622 million confirmed COVID-19 cases, including over 6.5 million deaths [1]. Some populations have been disproportionally impacted by the pandemic, including refugees, asylum seekers, and migrants [2–8]. For instance, 2.5–3 fold increases in the risk of COVID-19 infection and 3–4 times higher COVID-19 fatality rates have been reported for such communities in comparison to national averages [3, 9]. The existing challenges facing such vulnerable populations, such as crowded living conditions, co-morbidities, financial hardship, and past experiences of stigma or prejudice are further compounded by the impact of COVID-19. Undocumented migrants also are particularly vulnerable with poor access to healthcare and potentially unsafe living and working conditions [2–8].

International organizations have emphasized the importance of including vulnerable populations in COVID-19 vaccination efforts. For instance, WHO and other organizations, such as the European Centers for Disease Control (ECDC), have stressed that refugees, asylum seekers, and undocumented migrants should have equitable access to COVID-19 vaccination regardless of legal status [10, 11]. Despite such urgings from international organizations, it is unclear how many of the estimated 12.8 billion vaccine doses administered across the globe [1] have been provided to such populations.

There are also gaps in the literature on perceived acceptance to COVID-19 vaccinations in refugee and migrant populations and a need for more evidence concerning the factors influencing decisions to get vaccinated, such as access to information and educational resources and opportunities to have questions answered. Past review articles on COVID-19 vaccination and such populations have focused solely on Europe [12, 13] or Latin America [14], or predominantly focused on ethnic minorities or broadly defined migrants [15] or addressed uptake and acceptance of vaccines in general [12]. In this rapid review, we captured the current landscape regarding acceptance of and access to COVID-19 vaccinations for refugee, asylum seeker, and undocumented migrant populations globally. We focused on these populations as being particularly vulnerable to COVID-19 and lacking adequate prioritization in health policies around COVID-19 vaccination allocation. This rapid review provides the basis to highlight best practices for the critical contributions of COVID-19 vaccinations to the end or control the pandemic.

## Methods

### Search Strategy

**FIGURE 1:**
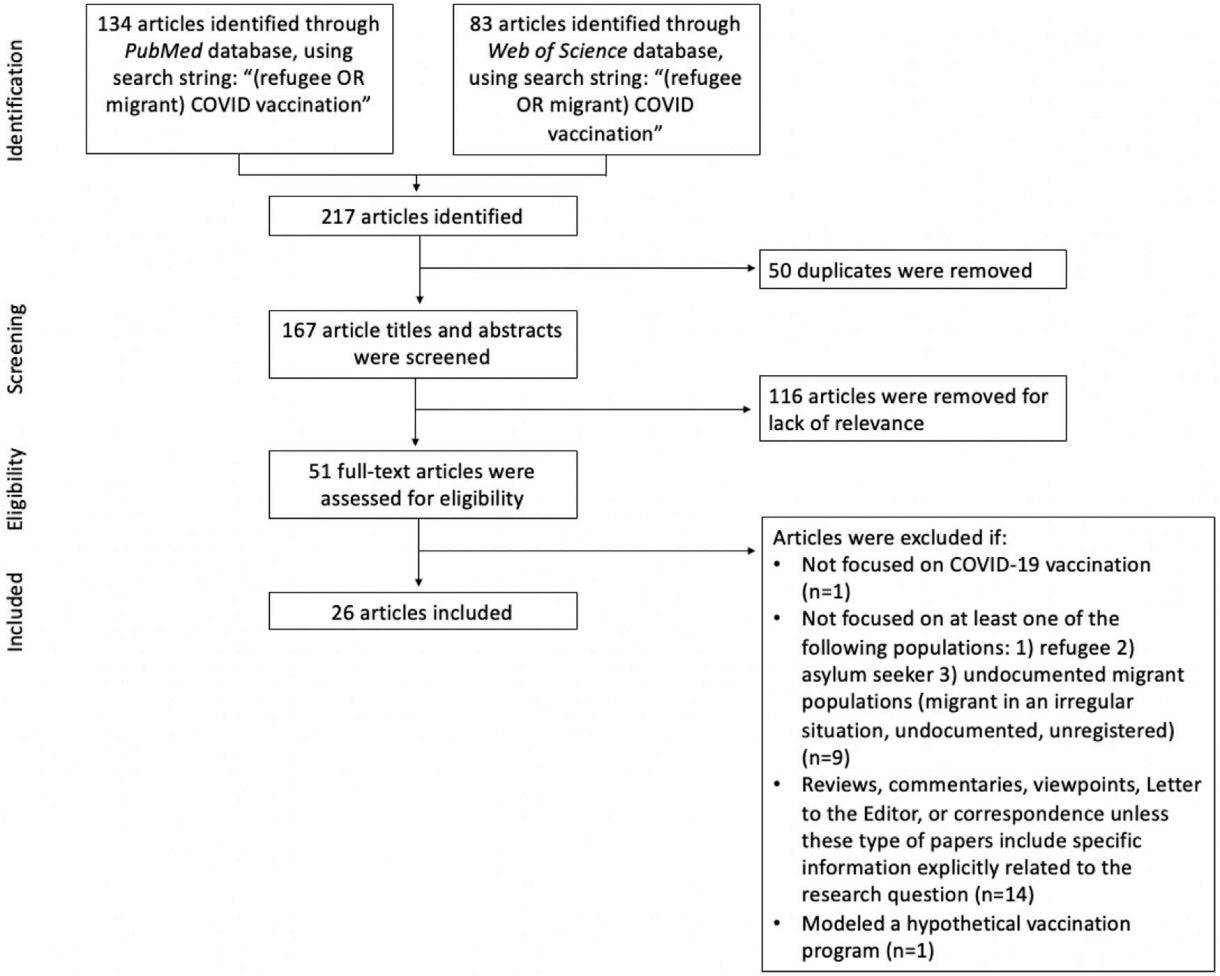
Search flow diagram. Database searches were conducted in June 2022. (Greece, 2022).

We conducted a rapid review of the literature to assess the level of access to COVID-19 vaccinations for refugees, asylum seekers, and undocumented migrants, by identifying facilitators and barriers, such as examining vaccine acceptance among these populations due to their beliefs and perceptions [16, 17]. See Figure 1 and Supplementary File S1 for overall search process including the inclusion and exclusion criteria for this rapid review [16, 17] based on PRISMA guidelines [18], to provide an update on the current literature in light of the recent refugee crises’ developments.

### Data Extraction and Analysis

Data extraction was initially performed by two research team members (AAN and ZP) and then reviewed by a third research team member (EK) to ensure quality data selection. For each research study, Supplementary File S2 describes the study population, methods, geographic location, and time period. While reviewing the full-text articles, we documented emergent themes that reflected specific factors of COVID-19 vaccine acceptance and access for global refugees, asylum seekers, and undocumented migrants in the studies.

## Results

### Overview

A total of 26 articles published between 2021 and 2022 were eligible for this review, including qualitative and quantitative research studies (*n* = 13) and commentaries (*n* = 13). Of the 13 research studies, 7 were quantitative surveys, 2 qualitative semi-structured interviews, 1 qualitative focus groups, and 3 mixed methods.

### Reported Levels of COVID-19 Vaccine Acceptance

The majority of studies reported results of COVID-19 vaccine acceptance by refugees, asylum seekers and undocumented migrants from over 15 host countries. High rates of vaccine acceptance were reported in some studies of refugee populations from a single origin country and single host country location. For instance, a study of 926 Venezuelan migrants in Colombia revealed high levels of COVID-19 vaccine acceptance, with intent to be vaccinated ranging from 74.7% to 91.6% across different study sites [19]. Similarly, a survey of 306 Mexican migrants deported from the U.S. found that 70.1% had accepted COVID-19 vaccination (at least one dose), and of those not yet vaccinated, 32.5% indicated they would accept vaccine if offered [20]. In addition, study of 1037 Syrian refugees in Lebanon found that most (66%) refugees intended to get vaccinated if the vaccine was free and safe [21].

A study of 360 temporary foreign workers from a single origin country (Bangladesh) was carried out across multiple host countries including mainly Arab states of the Persian Gulf (67%) and Singapore/Malaysia (23%) [22]. Here, the authors found that overall vaccine hesitancy was low (25%), although undocumented temporary foreign workers (N = 41) had higher levels of vaccine hesitancy (41%) than those with valid visas (N = 218) (22%). In addition, at the world’s largest refugee camp in Cox’s Bazar, Bangladesh, a high degree of acceptance of offered vaccination was evident among Rohingya refugees, with more than half of the eligible population fully vaccinated in 4 months [23]. A high rate of COVID-19 vaccination acceptance was also found in a U.S. study of 435 refugees from a variety of origin countries, where over 70% of refugees indicated intent to get vaccinated [24]. Similarly, in a study of 516 refugee and asylum seekers in Australia, 88% were unvaccinated, and 71.9% (N = 314) of those unvaccinated were intending to get vaccinated [25].

Lower rates of COVID-19 vaccine acceptance were seen in some of the other studies. A large multi-country study of 1,914 forcibly displaced persons in Peru, Colombia, Brazil, Venezuela, Turkey, Jordan, Uganda, and the Democratic Republic of Congo revealed highly variable rates of COVID-19 vaccine acceptance across the host countries, with some being quite low, although the overall acceptance rate was still 64% [7]. Low (41.2%) demand for COVID-19 vaccine was reported in one study of undocumented migrants (N = 812) in the U.S. and Europe who originated from various countries [26]. In addition, two small studies also reported relatively high COVID-19 vaccine hesitancy among refugees, asylum seekers, and undocumented migrants (42.6% and 72%) [27, 28]. Interestingly, two studies [20, 28] reported increases in vaccine acceptance attitudes over time and as vaccine became available.

### Factors Influencing COVID-19 Vaccine Acceptance

#### Access to Accurate Information From Trusted Source

Many of the studies cited the lack of access to accurate, understandable information from trusted sources as barriers to COVID-19 vaccine acceptance by refugees, asylum seekers, and undocumented migrants [5, 7, 19–21, 23, 25–31]. For instance, one large study reported lack of clear information about COVID-19 vaccine and its safety as the most common barrier (54.5%) to vaccine acceptance among such survey participants [25]. Korobkova et al. found 45% of respondents reported receiving no information about COVID-19 vaccine eligibility, safety or costs from their host country’s health authorities [7]. Instead, study participants reported using sources such as social media (40%), television (25%), and family and friends (24%) to learn about COVID-19 vaccine information. Across several studies, social media and misinformation were found to lead to a variety of erroneous personal beliefs such as COVID-19 was a “hoax or Western disease” or the COVID-19 vaccine contains a microchip or alters DNA and other conspiracy theories [29, 30].

COVID-19 vaccines recency was a factor in vaccine hesitancy [19, 21, 26, 28]. For instance, Page et al. reported that 77.3% of participants were positive about vaccinations in general, but only 56.5% were positive about the new COVID-19 vaccines [26]. Also over 51% of the undocumented Venezuelan migrants in the large Colombia study felt the COVID-19 vaccine being offered was too new and that social media made them unsure about it [19].

Language barriers were another obstacle [7, 20, 29, 31]. Crawshaw et al. highlighted the need for accurate information to be clearly conveyed in a manner and language appropriate for the target population and co-developed with community representatives for COVID-19 vaccination programs [29]. Another study cautioned that undocumented migrants may be at risk of digital exclusion [5].

#### Influence of Gender or Cultural/Geographic Origins

Some studies highlighted gender-based differences in COVID-19 vaccine acceptance within refugee, asylum seeker, and undocumented migrant populations [19, 23, 26]. However, it does not seem there is a clear weight of evidence supporting one gender versus another. In the large multi-country study of such populations carried out by Page et al., women were found to be more likely than men to endorse vaccination [26]. Concerns about COVID-19 vaccination during pregnancy were reported as “very common” in the study of Venezuelan refugees in Colombia [19]. In addition, being male was significantly associated with higher odds of intending to receive a COVID-19 vaccine in a large US study [24]. However, other studies found no gender differences. For instance, the large study of Syrian refugees in Lebanon reported no difference between men and women in vaccine acceptance [21].

Two studies highlighted differences in vaccine acceptance attitudes based on cultural or geographic origins of refugees, asylum seekers, and undocumented migrants. A study of such individuals in settlements in Rome found greater vaccination hesitancy in those from sub-Saharan Africa and Eastern Europe [32]. Shaw et al. also found that vaccine hesitancy of refugees could be related to concerns that the vaccine was prohibited by their religion [28].

#### Trust in Host Country Government

A few studies emphasized trust in host countries’ governments as an important factor in promoting vaccine acceptance [21, 25, 33]. These studies highlighted the lack of trust of local health authorities as a significant barrier to COVID-19 vaccine acceptance. Fear of discrimination and stigma when interacting with host countries’ health systems was also raised as a barrier. Involvement of trusted community leaders was proposed as a way to address such barriers and increase trust in host country government programs.

### Reported Levels of COVID-19 Vaccine Uptake

COVAX, an international initiative co-led by the Coalition for Epidemic Preparedness Innovations (CEPI), Gavi and WHO, with UNICEF as a key delivery partner, was created to accelerate COVID-19 vaccine development and manufacture and to address the issue of equitable global vaccine access [34]. As of 1 September, 2022, 4.5 billion doses have been administered across COVAX participating countries [34]. COVAX also established a humanitarian buffer, approximately 5% of total available doses, to try to provide COVID-19 vaccine access for vulnerable individuals in humanitarian situations, which would include refugees, asylum seekers, and undocumented migrants. The COVAX initiative [34], was brought up several times as a mechanism that could provide COVID-19 vaccine access to such populations but had fallen short of its potential [7, 33, 35]. Ibrahim et al. commented on how the COVAX initiative did not explicitly stipulate the inclusion of refugees in its distribution guidelines, which would impact equitable vaccine distribution in regions such as Africa, where vaccines supplies are limited and numerous large refugee populations exist [35]. COVAX’s small humanitarian buffer was discussed as a potential way to provide access to refugees [7, 33].

Despite such efforts, access of refugees, asylum seekers, and undocumented migrants to COVID-19 vaccinations was reported to have varied considerably from country to country and over time as vaccine availability and prioritizations evolved. There was no study in the sample that documented the exact level of coverage for such populations within a country. Multiple countries put in place policies that would include refugees, asylum seekers, and undocumented migrants in vaccine roll-out efforts, but often implementation and/or monitoring of such efforts were lacking.

One single-country quantitative study involved 306 Mexican migrants deported from the U.S [20]. The authors found 70.1% of study participants had received at least one COVID-19 vaccine dose, and 99.9% had been provided the vaccine while in the U.S. A multi-country quantitative study included 1,914 forcibly displaced people in Peru, Colombia, Brazil, Venezuela, Turkey, Jordan, Uganda, and the DRC [7]. Respondents in Peru (32%), Brazil (51%), and Jordan (38%) indicated that their host country gave them temporary legal status to allow access to any services. The authors mentioned that the Jordanian government adapted existing vaccination and healthcare measures provided to asylum seekers and refugees to include COVID-19 needs. Korobhkova et al. also mentioned that the Ugandan government policy identified refugees as a priority and targeted them in their vaccine roll-out. Despite these factors, only one refugee in Uganda reported receiving COVID-19 vaccine out of all the respondents included in their study [7].

The commentary by Emeka et al. highlighted that vaccine access for Nigeria was distributed through COVAX, providing an estimated 4 million doses in March 2021, and refugees were among the prioritized groups in accessing the vaccines, but no estimate on the level of vaccine coverage of refugees was provided [36]. Another commentary piece [37], described how the COVID-19 vaccination schema in Rwanda included refugees if they met any of the standard criteria of being in a high-risk group and documented that in March 2021, and 412 refugees were vaccinated but did not explain why this number was so low.

These sparse examples highlight the paucity of reliable data on refugee, asylum seeker, and undocumented migrant access of COVID-19 vaccinations, a topic also discussed in several of the included commentaries [26, 31, 33]. These data deficiencies make it difficult to assess the level of access to COVID-19 vaccinations for these populations, tailor vaccination programs to specific refugee, asylum seeker, and undocumented migrant populations, and to provide continuation of care, such as appropriately timed subsequent doses.

### Factors Determining Access to COVID-19 Vaccination

Buonsenso and Both [31] and [38] reported positive experiences with active vaccination programs for refugees and asylum seekers in Germany, Nepal, Jordan and Rwanda [31]. However, most papers documented a range of barriers and concerns regarding access to COVID-19 vaccination for refugees, asylum seekers and undocumented migrants. These included concerns around exclusion from or de-prioritization in national vaccine roll-out efforts. Undocumented migrants in particular indicated concerns regarding vaccination cost and potential to experience immigration checks and potential deportation.

#### Conflicting Vaccination Prioritization Between International and National Authorities

A patchwork of policies globally was found, as countries developed prioritization guidelines and vaccine distribution plans for COVID-19 vaccines. Manirambona et al. [37] highlighted the tension that exists between international and national policies, with organizational bodies such as the WHO, UNHCR, and UNICEF providing recommendations pertaining to refugee, asylum seeker, and undocumented migrant populations access to COVID-19 vaccinations yet relying on national actors to coordinate successful delivery of vaccination programs. Korobkova et al. referenced a United Nations High Commissioner for Refugees (UNHCR) April 2021 report to highlight how only 20 countries were known to have begun vaccinating refugees and asylum seekers on an equal footing to citizens, despite other evidence that approximately 100 countries had officially included refugees or asylum seekers in their national vaccination deployment plans or local distribution policies [7, 33].

The viewpoint article by Armocida et al. highlighted the considerable variation in COVID-19 vaccination policy across European Union member states (EU MSs) despite the urgings by the European Centre for Disease Control (ECDC) and other European institutions for EU MSs to include migrants and refugees as potential target populations for vaccination [39]. Crawshaw et al. [29] and [39], commented on how some European governments (e.g., Spain, Netherlands, UK, France, Italy) removed healthcare access barriers during the pandemic to allow for non-residents to receive COVID-19 testing and vaccination. The inclusive policies of the countries of Germany, Jordan and Uganda were also held as exemplary with regard to COVID-19 vaccine access for non-citizens [7, 31, 35, 38]. Tiirinki et al., 2022, highlighted the policy in Finland, where central government COVID-19 vaccination policy would include refugees, asylum seekers and undocumented migrants, but it was left to the municipalities to implement and no systematic data were collected to assess equitable access [40]. Substantial variation in the extent of prioritization and inclusion of such populations in COVID-19 vaccination policy and program implementation across the high-income countries of the U.S., Canada, Australia, Japan and South Korea was also highlighted in another review [8]. Two articles highlighted the Lebanese Ministry of Public Health’s National Deployment and Vaccination Plan for COVID-19 vaccines, which included non-citizens, yet the authors were skeptical of the level of actual access provided [9, 33]. These reservations were based on xenophobia on social media by some Lebanese political actors [33], and statistics such as only 12.5% of Syrian refugee children in Lebanon are up to date on routine vaccinations [9]. These are just a couple of examples of how despite policy inclusion of refugees, asylum seekers, and undocumented migrants, limited data of actual COVID-19 vaccination coverage exists.

The level to which governments made refugees, asylum seekers, and undocumented migrants aware of policies regarding vaccine access also varied from nation to nation. Page et al. found that 86.4% of undocumented migrants (N = 812) distributed across Switzerland, USA, Italy and France perceived COVID-19 vaccination to be accessible in their host country [26]. Other studies, such as [27], highlighted perceptions that migrant communities would be excluded from or de-prioritized in COVID-19 vaccine roll-out despite government policies including migrants and refugees as named populations.

#### Lack of Status or Legal Documentation

Several studies noted that the lack of health insurance in the host country as a significant perceived barrier for refugees, asylum seekers, and undocumented migrants in accessing COVID-19 vaccination, especially for undocumented migrants [7, 26, 27, 31, 33]. For undocumented migrants, fear of immigration checks and deportation can be a significant barrier to vaccination when the vaccination programs are run by local governments or require proof of documentation, even when health authorities state that COVID-19 vaccines would be provided without immigration checks. Buonsenso and Both [31], echoed this concern and a proposed solution was to have policymakers agree to include an anonymous code for undocumented migrants who preferred to not be officially registered in national vaccination registries.

#### Physical and Financial Barriers

Both physical and financial barriers can make it difficult for refugees, asylum seekers, and undocumented migrants to access COVID-19 vaccination sites, which are often found in city centers that might not be convenient or feasible to reach for specific subpopulations. Specifically, [32], highlighted the difficulties that elderly subpopulations might face when attempting to physically get to and access vaccination centers. In terms of the financial barriers, both Zard et al. 2021 and Buonsenso and Both, 2022 commented on how perceived costs of COVID-19 vaccination and actual costs, such as out-of-pocket expenses (e.g., transportation, lost wages), might lead to refugees, asylum seekers, and undocumented migrants not accessing vaccination [31, 33]. In the 2021 study by Deal et al. [27], undocumented migrants, in particular, had concerns about being charged for vaccination, even though the national health authorities had stated that COVID-19 vaccines would be free of charge.

#### Outreach Programs

Increasing outreach efforts to engage refugees, asylum seekers, and undocumented migrants was the most common factor cited to positively influence access to COVID-19 vaccination. In a very large study in western U.K of 126,710 individuals categorized as “non-English-speaking, minority ethnic groups, refugees and asylum seekers,” trends were reported of increased vaccination aligning with increased outreach efforts [41]. These outreach efforts included use of trusted community spaces such as community centers, mosques, churches, supermarkets, etc., and involved written materials and social media outputs generated in appropriate languages for the target communities. Other studies also encouraged actively engaging national and international non-government organizations (NGOs) rather than government entities to deliver COVID-19 vaccinations to refugees, asylum seekers, and undocumented migrants [31].

Another positive example of the impact of outreach efforts on refugee COVID-19 vaccine uptake was the program implemented for Rohingya refugee community in the Cox’s Bazar camp in Bangladesh [23]. Engagement with refugee community leaders about low rates of COVID-19 vaccination in women led to development of educational programs designed to combat false rumors, women-only radio listener’s clubs, religious group-study sessions, and use of female vaccinators to approach refugee women. These efforts resulted in a high level of vaccination acceptance among women with greater than 80% in the target group being vaccinated in the first month of the initiative [23].

The differing challenges of outreach efforts with refugee, asylum seeker, and undocumented migrant populations in organized camps or identified settlements rather than dispersed in urban or rural settings was mentioned in a number of papers. While overall COVID-19 vaccine acceptance was high in the Syrian refugee population studied in Lebanon [21], refusal to get vaccinated was higher among refugees living outside informal tented settlements compared to those living inside. The authors suggested this reflected the relative ease with which humanitarian programming can reach refugees inside organized areas in contrast to “hard-to-reach” refugees dispersed in urban and rural locations.

Several articles recommended a variety of outreach efforts to improve refugee, asylum seeker, and undocumented migrant access to COVID-19 vaccine through mobile efforts to exchange reliable information and address physical or location challenges [24, 28, 30, 31, 39]. For instance, participants in the Mahimbo et al. 2022 study suggested outreach efforts to improve vaccine access and uptake such as mobile vaccination clinics and “peer-to-peer story sharing” to engage within their own communities to increase trust in the vaccines [30].

## Discussion

The results of our rapid review identified a breadth of both quantitative and qualitative data describing the levels of acceptance and access to COVID-19 vaccination among refugees, asylum seekers, and undocumented migrants globally.

Overall, there were differences in willingness and acceptance of COVID-19 vaccination and limited access to vaccination across many countries hosting refugees, asylum seekers, and undocumented migrants. While some of studies documented high or mixed COVID-19 vaccine hesitancy in these populations [7, 26–28], others found relatively high COVID-19 vaccine acceptance rates [21, 22, 24, 25], with some examples even of higher acceptance rates in these populations than those found in the respective host country population or documented migrants [21, 22]. However, while vaccine hesitancy was not found to be a consistent major barrier to achieving high vaccination rates in refugee, asylum seeker, and undocumented migrant populations, the majority of study authors indicated much more needed to be done to achieve high levels of COVID-19 vaccination coverage in these highly vulnerable populations. Studies reporting the increase in vaccination of refugees, asylum seekers, and undocumented migrants as a result of trust building efforts and community outreach activities [23, 41], have clear implications for improvements in COVID-19 vaccination policy and implementation strategies relative to these populations. There were two core impactful approaches discussed. First, the importance of involvement of community leaders and trusted individuals in dissemination of accurate information and in appropriate language and cultural context. Second, ease of access to vaccination with provision of pop-up vaccination clinics in close proximity to the target refugee, asylum seeker, and undocumented migrant population to alleviate trust concerns and minimize cost (transportation costs or lost income due to time taken to get vaccinated). A good example of governments addressing the importance of community-based outreach, is the Health Resources and Services Administration (HRSA) funding to train community workers in the United States [42].

While lack of trust in host government authorities and fears of deportation can be barriers, other barriers are reported relative to the host country, including general population xenophobia and discrimination relative to refugees, asylum seekers, and undocumented migrants [7]. Efforts to counter negative social media against such populations and anti-vaccine misinformation will also need to be important components of public health efforts to achieve high level COVID-19 vaccination coverage in these populations.

One of the obvious underlying causes of lack of priority or poor implementation of COVID-19 vaccination of refugees, asylum seekers, and undocumented migrants in a particular country is the lack of equity of distribution of COVID-19 vaccines globally. While COVAX initiative has attempted to ease supply of vaccine to low income countries, several of which have large numbers of refugees (e.g., Uganda or Jordan), the political leaders in countries are often forced to make politically difficult decisions due to cost and limited vaccine supply with regard to prioritizing refugees, asylum seekers, and undocumented migrants relative to citizens which can fundamentally impact protection of these highly vulnerable populations.

Lastly, while most governments endorsed COVID-19 vaccination policies that include, or at least do not exclude, refugees, asylum seekers, and undocumented migrants within their country, evidence of successful implementation of these programs appears to be lacking in most instances. This important gap makes it difficult to assess extent of vulnerability of populations and to better tailor and implement COVID-19 outreach programs.

The importance of acting on the findings and recommendations described here for COVID-19 vaccination is growing given increases in refugee, asylum seeker, and undocumented migrant populations worldwide and many recommendations are equally important for vaccine preventable diseases in general, and responses to other emerging diseases. UNHCR estimates that total number of people across the globe who were forced to flee their homes now exceeds 100 million people [43]. It is also estimated that the global refugee population has reached a new high in 2022, surpassing 30 million people [44]. Europe is currently seeing the largest migration this century as a result of the Russian invasion of Ukraine. As of April 25, 2022, over 5.2 million Ukrainians have left the country, with almost 3 million taking refuge in Poland [44]. Low COVID-19 vaccination rates have been reported in Poland, and were even lower in Ukraine, suggesting these refugees are likely particularly vulnerable [13].

### Strengths and Limitations

A key strength of our study was the inclusion of refugee, asylum seeker, and undocumented migrant studies across the globe. We specifically included undocumented migrants in the review so as to capture the unique barriers they experience, such as lack of documentation or increased levels of mistrust of public health authorities. One limitation was that there were differences in the participant sample sizes across the different studies, which poses a challenge in comparing the results. Another limitation was the lack of inclusion of the non-peer reviewed literature.

### Conclusion

In conclusion, this review identified several key areas for improvement of COVID-19 vaccination access and acceptance for global refugee, asylum seeker, and undocumented migrant populations. The global community needs to encourage improvements in government policy, program implementation and assessment regarding COVID-19 vaccination of these populations. While refugee, asylum seeker, and undocumented migrant inclusive policies are in place in many countries, almost all countries fail to document successful implementation of those policies and data are lacking to identify areas for improvement. In addition, there is increasing need to improve trust of authorities and expand efforts to reduce fear, particularly for undocumented migrants. Further research is needed on how to achieve equitable access to COVID-19 and other life-saving vaccines for refugees, asylum seekers and migrants.

A particularly important impactful area for increased COVID-19 vaccination coverage among refugees, asylum seekers, and undocumented migrants is the provision of accurate information in appropriate language, particularly to counter misinformation *via* social media. Such misinformation often fuels public xenophobia and exacerbates fears of deportation for migrants lacking legal status. Outreach and community engagement efforts need to be strongly supported to get reliable information to the target refugee, asylum seeker, and undocumented migrant communities and to provide ease of access to offered vaccination. Ensuring equitable distribution of COVID-19 vaccines to countries across the globe and equity in access to vaccination for everyone within these countries, regardless of legal status, remains a vital goal. Pandemics do not recognize country borders, and with the likelihood of more future pandemics, as well as more refugees from political unrest, economic hardships, or climate change, a global agenda should be put forward to prepare countries regarding vaccine access to the refugee and migrant populations as the most vulnerable in society. The WHO and other UN agencies should work closely with governments to provide resources, research, and help to develop better strategies to address vaccination of these vulnerable populations during pandemics. The findings and recommendations presented here make a valuable contribution towards addressing these increasingly pressing problems and should help improve future policy and program implementation.
