# Supplementary material for: Rapid Review of COVID-19 Vaccination Access and Acceptance for Global Refugee, Asylum Seeker and Undocumented Migrant Populations
Source: Int J Public Health. 2022 Dec 22;67:1605508. doi: 10.3389/ijph.2022.1605508 (PMC9812946; doi:10.3389/ijph.2022.1605508)
Supplement: Supplementary file 1 [file DataSheet2.docx]

**Supplemental File 2**. Descriptions of included articles which are listed in order of appearance in the paper (Greece, 2022).

| **Reference number** | **Authors** | **Article Title** | **Article Type** | **Host Countries/Regions** | **Origin Countries/Regions** | **Study Methods** | **Study Time Period** | **Study Participants (n)** |
| --- | --- | --- | --- | --- | --- | --- | --- | --- |
| 5 | Knights et al., 2021 | ﻿Impact of COVID-19 on migrants' access to primary care and implications for vaccine roll-out: A national qualitative study | Qualitative study | United Kingdom | Multiple | Semi-structured qualitative interviews | July -November 2020 | 17 |
| 7 | Korobkova et al., 2022 | ﻿Left Behind: The Multiple Impacts of Covid-19 on Forcibly Displaced People | Quantitative study | Peru, Colombia, Brazil, Venezuela, Turkey, Jordan, Uganda, DRC | Multiple | Multi-country survey | 25 April - 9 May 2021 | 1,914 |
| 8 | Berardi et al., 2022 | A vicious cycle of health (in)equity: Migrant inclusion in light of COVID-19 | Documentary analysis | United States, Australia, Canada, Japan, South Korea | Not specified | N/A | N/A | N/A |
| 9 | Jawad et al., 2021 | ﻿Refugee access to COVID-19 vaccines in Lebanon | Correspondence | Lebanon | Not specified | N/A | N/A | N/A |
| 19 | Benavides-Melo et al., 2021 | COVID-19 vaccination intention among Venezuelan migrant populations in Colombia, 2021 | Letter to the Editor | Colombia | Venezuela | Three random sample-based community surveys | Late August - early September 2021 | N/A |
| 20 | Martínez-Donate et al., 2022 | COVID-19 testing, infection, and vaccination among deported Mexican migrants: Results from a survey on the Mexico-U.S. border | Quantitative study | United States | Mexico | Cross-sectional quantitative survey | August 2020 - July 2021 | 306 |
| 21 | Salibi et al., 2021 | COVID-19 vaccine acceptance in older Syrian refugees: Preliminary findings from an ongoing study | Quantitative study | Lebanon | Syria | ﻿Phone interview surveys | January - February 2021 | 1037 |
| 22 | West et al., 2021 | ﻿COVID-19 Vaccine Hesitancy among Temporary Foreign Workers from Bangladesh | Quantitative study | Arab states of the Persian Gulf, Singapore, Malaysia | Bangladesh | Observational study | January - February 2021 | 41 |
| 23 | Alam, 2022 | ﻿Vaccinating women against COVID in world's largest refugee camp | Correspondence | Bangladesh | Myanmar | N/A | N/A | N/A |
| 24 | Zhang et al., 2021 | ﻿Acceptance of COVID-19 Vaccine Among Refugees in the United States | Quantitative study | United States | Multiple | Online surveys | December 2020 - January 2021 | 435 |
| 25 | Liddell et al., 2021 | Factors associated with COVID-19 vaccine hesitancy amongst refugees in Australia | Quantitative study | Australia | Multiple | Online surveys | 2 June 2021-2 July 2021 | 516 |
| 26 | Page et al., 2022 | COVID-19 vaccine hesitancy among undocumented migrants during the early phase of the vaccination campaign: a multicentric cross-sectional study | Quantitative study | Switzerland, United States, Italy, France | Multiple | Questionnaire | Mid-February - late May 2021 | 812 |
| 27 | Deal et al., 2021 | Strategies and action points to ensure equitable uptake of COVID-19 vaccinations: A national qualitative interview study to explore the views of undocumented migrants, asylum seekers, and refugees | Qualitative study | United Kingdom | Multiple | Semi-structured qualitative interviews | September 2020 - March 2021 | 30 |
| 28 | Shaw et al., 2022 | COVID-19 vaccination intention and behavior in a large, diverse, US refugee population | Mixed methods study | United States | Central and East Africa, Southeast Asia, the Middle East | Quantitative survey and semi-structured qualitative interviews | December 2020 - March 2021 | 244 |
| 29 | Crawshaw et al., 2021 | What must be done to tackle vaccine hesitancy and barriers to COVID-19 vaccination in migrants? | Perspective | Not specified | Not specified | N/A | N/A | N/A |
| 30 | Mahimbo et al., 2022 | ﻿Factors influencing refugees' willingness to accept COVID-19 vaccines in Greater Sydney: a qualitative study | Qualitative study | Australia | Multiple | Focus group interviews | April and May 2021 | 37 |
| 31 | Buonsenso and Both, 2022 | ﻿Ensuring global access to COVID-19 vaccines: deployment strategies for refugees and migrants must not be forgotten | Correspondence | Not specified | Not specified | N/A | N/A | N/A |
| 32 | Bentivegna et al., 2022 | ﻿Access to COVID-19 Vaccination during the Pandemic in the Informal Settlements of Rome | Mixed methods study | Italy | Multiple | Qualitative data collection and quantitative data collection | 1 June 2021 - 30 September 2021 | 160 |
| 33 | Zard et al., 2021 | ﻿Leave no one behind: ensuring access to COVID-19 vaccines for refugee and displaced populations | Comment | Not specified | Not specified | N/A | N/A | N/A |
| 35 | Ibrahim et al., 2021 | ﻿Will refugees also get vaccinated against COVID-19 in Africa? | Commentary | Africa | Not specified | N/A | N/A | N/A |
| 36 | Emeka et al., 2022 | COVID-19 vaccine roll-out at the community level in developing countries: Lessons learnt from Cross River State, Nigeria | Commentary | Nigeria | Cameroon | N/A | N/A | N/A |
| 37 | Manirambona et al., 2021 | COVID-19 Vaccines: Ensuring Social Justice and Health Equity among Refugees in Africa | Viewpoint | Africa | Not specified | N/A | N/A | N/A |
| 38 | Bellizzi et al., 2020 | Vaccination for SARS-CoV-2 of migrants and refugees, Jordan | Editorial | Jordan | Syria, Palestine, Iraq, Yemen, Sudan | N/A | N/A | N/A |
| 39 | Armocida et al., 2021 | Challenges in the equitable access to COVID-19 vaccines for migrant populations in Europe | Viewpoint | Europe | Not specified | N/A | N/A | N/A |
| 40 | Tiirinki et al., 2022 | COVID-19 in Finland: Vaccination strategy as part of the wider governing of the pandemic | Documentary analysis | Finland | Not specified | N/A | N/A | N/A |
| 41 | Berrou et al., 2022 | Leaving No One Behind: Interventions and Outcomes of the COVID-19 Vaccine Maximising Uptake Programme | Mixed methods study | United Kingdom | Multiple | Retrospective descriptive cohort study, semi-structured qualitative interviews | May - June 2021 | 235 |
