# Supplementary material for: Rapid Review of COVID-19 Vaccination Access and Acceptance for Global Refugee, Asylum Seeker and Undocumented Migrant Populations
Source: Int J Public Health. 2022 Dec 22;67:1605508. doi: 10.3389/ijph.2022.1605508 (PMC9812946; doi:10.3389/ijph.2022.1605508)
Supplement: Supplementary file 2 [file DataSheet1.docx]

**Supplemental File 1.** Details of database searches and selection process for final sample. (Greece, 2022)

Database searches were conducted in PubMed and Web of Science using search string “(refugee OR migrant) COVID vaccination”. Database searches were not limited by article publication date because searches included “COVID” as a term, which narrowed the resulting articles. Initial database searches yielded 134 articles from PubMed and 83 articles from Web of Science. Of the 217 articles total, 50 were identified as duplicates and were removed. 167 article titles and abstracts were manually screened, and articles were removed if identified as not relevant. 51 full-text articles were accessed for eligibility. Quantitative and qualitative research studies that provided primary data, or opinion articles that specifically discussed or provided relevant data on the topic of COVID-19 vaccination access and barriers and facilitators for RASUM populations were included. Articles were excluded if: not focused on COVID-19 vaccination (n=1); not focused on at least one of the following populations: 1) refugee 2) asylum seeker 3) undocumented migrant populations (e.g., migrant in an irregular situation, undocumented, unregistered in a country different to their home country) (n=9); were reviews, commentaries, viewpoints, Letter to the Editor, or correspondence, unless these type of papers include specific data explicitly related to the research question (n=14); or modeled a hypothetical vaccination program (n=1). Of the 51 full-text articles assessed, 26 were included in the final sample.
